# Multiple sclerosis in the far north - incidence and prevalence in Nordland County, Norway, 1970–2010

**DOI:** 10.1186/s12883-014-0226-8

**Published:** 2014-12-04

**Authors:** Espen Benjaminsen, Johnny Olavsen, Merethe Karlberg, Karl B Alstadhaug

**Affiliations:** Department of Neurology, Nordland Hospital Trust, Post box 1480, 8092 Bodø, Norway; Nordland Hospital Trust, Vesterålen, Norway; Institute of Clinical Medicine, University of Tromsø, Tromsø, Norway

**Keywords:** Multiple sclerosis, Norway, Epidemiology, Prevalence, Incidence

## Abstract

**Background:**

The risk of multiple sclerosis (MS) increases with increasing latitude. Taking into consideration that Norway has a large latitude range, a south-to-north gradient would be expected. However, previous studies have reported an uneven distribution of the disease in Norway, with a relatively low prevalence in the most northern parts of the country.

We describe the incidence and prevalence of MS in a county in the north of Norway over a period of 40 years.

**Methods:**

All patients with MS living in Nordland County in the period 1970–2010 were identified by reviewing hospital charts. The patients were included if they met the criteria of definitive or probable MS according to Poser [Ann Neurol 13:227-231, 1983] or MS according to McDonalds [Ann Neurol 50:121-127, 2001]. Point prevalence at the beginning of the decades was calculated. The average annual incidence was calculated for 5-year periods.

**Results:**

The total crude prevalence on January 1, 2010 was 182.4 per 100 000. The annual incidence continuously increased from 0.7 per 100 000 in 1970 – 1974 to 10.1 per 100,000 in 2005 – 2009. The time delay from the first symptom to diagnosis was stable from 1975 to 2010. The proportion of primary progressive MS in the prevalence numbers was 38.2% in 1980, and decreases continuously, to 18.6% in 2010. The female to male prevalence ratio has been stable since 1990 at 2.2 to 1.

**Conclusion:**

The prevalence and the incidence of MS have steadily increased over a 40 year period. Nordland County is a high-risk area for MS.

## Background

**Figure 1:**
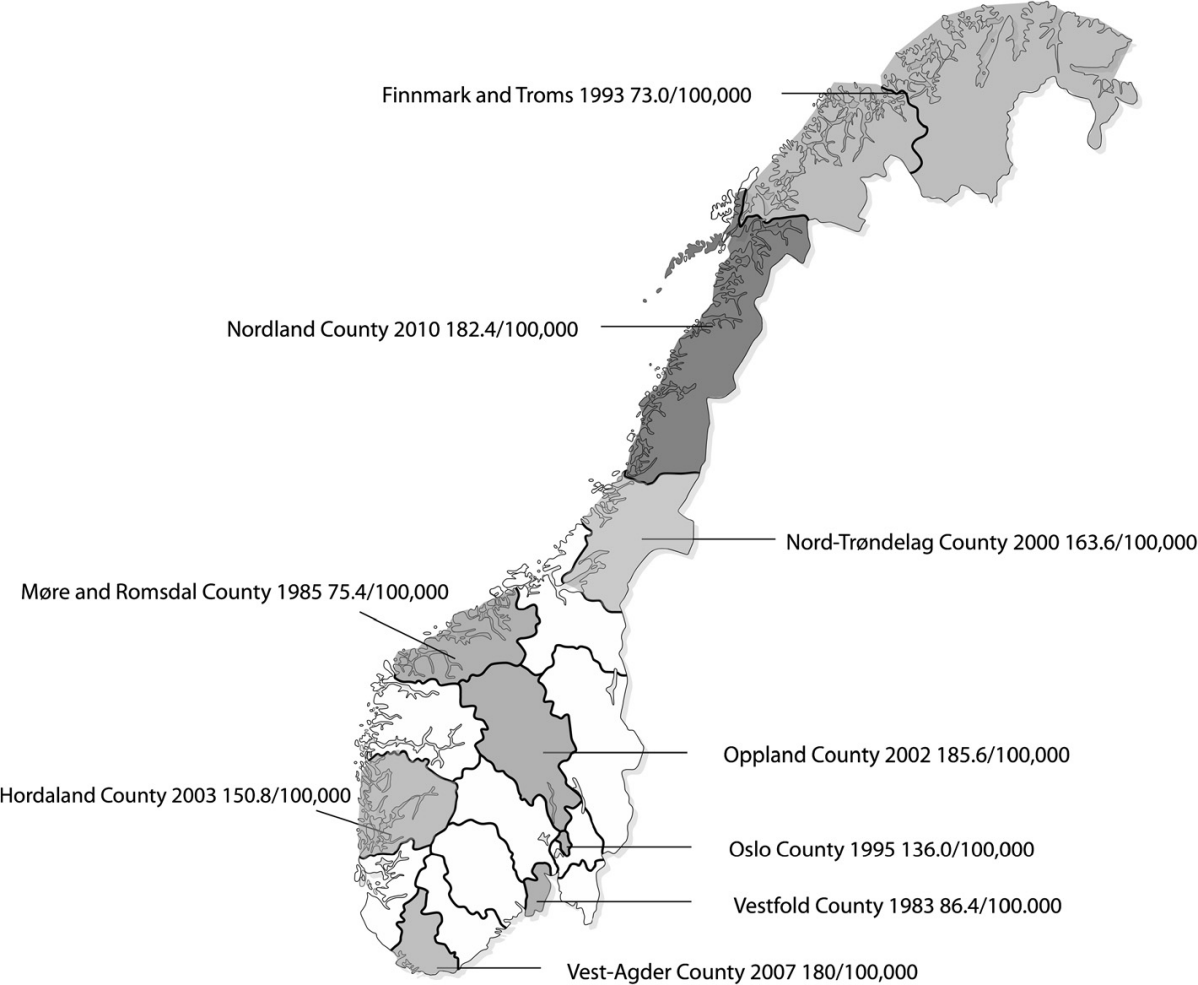
**Prevalence of MS in Norwegian counties.**

Multiple sclerosis (MS) is a chronic inflammatory demyelinating disease in the central nervous system with unknown aetiology. A latitude dependent gradient in the occurrence has been shown in many countries and regions. The risk of MS is increasing from south to north in Europe, North America and Japan [1-4] on the northern hemisphere, and from north so south in Australia and New Zealand [5,6] on the southern hemisphere. In general, the risk increases with increasing distance from the equator [7]. Norway has a long and narrow landscape, with the mainland stretching from 57° N to 71° N, and a similar north–south gradient of the MS prevalence would thus be expected. However, previous studies have shown an uneven distribution in Norway, with a relatively low prevalence in the most northern parts of the country (Figure 1) [8-15]. This variation has been attributed to specific genetic and environmental factors. There is accumulating evidence that hypovitaminosis D is an important risk factor for MS [16], and it has been suggested that low sun exposure, and thereby reduced production of cutaneous vitamin D, in the high latitudes could be one cause of the observed increase in these areas. The paradoxically low occurrence of MS in the north of Norway could then be explained by high dietary intake of vitamin D through fish consumption [17,18]. The genetics of the indigenous population of the far north could also be an explanation for the reversed gradient observed. The Sami people of northern Scandinavia seem to be partly protected against MS. The first known appearance among them is registered as late as the 1990s [10]. MS is associated with the human leukocyte antigen DRB1*15-DQB1*06, and the Sami have a low frequency of this haplotype [19].

To contribute to a more precise knowledge of the distribution of MS, we assess the prevalence and incidence of multiple sclerosis in Nordland County in the period 1970 to 2010. Interim data published in Norwegian in 2005, showed a prevalence of 105.6 per 100 000 at December 31, 1999 [20]. All data from this survey was reexamined. We describe trends in the occurrence over four decades in an area with a stable and relatively homogeneous population.

## Methods

This is a retrospective cross-sectional epidemiological study.

### Geographical area

Nordland is in the northern part of Norway, situated between latitude 64°56’ N and 69°20’ N. The Arctic Circle at 66°33’ N is running through the county in between Mo i Rana and Bodø, the two largest cities. The county, covering a total area of 38456 km^2^, includes the regions of Helgeland, Salten, Ofoten, and the islands of Lofoten and Vesterålen. The population at risk at January 1, 2010 was 236 271 (118537 men, 117734 women). The population was 243 179 in 1970, indicating an average yearly reduction in the population of 0.07% in the period.

The only neurological department is at the Nordland Hospital in Bodø, serving both outpatients and hospitalised patients. The department was founded in 1973, but a neurologist was at place a year earlier, and MS patients are registered from 1970. There are neurological outpatient services at the hospitals in Mosjøen (Helgeland) and Stokmarknes (Vesterålen).

### Case ascertainment

All patient with the diagnoses of MS treated at the hospital in Bodø were identified by searching for the diagnosis according to ICD 8 (340.08), ICD 9 (340) and ICD 10 (G35) in the hospital’s medical files. In addition to the files of Nordland Hospital in Bodø, we searched the files from the hospitals in Mosjøen and Stokmarknes. We also requested for files and cases from the neurological department in Namsos in the neighbor county to the south, and from the two nearest university hospitals, the University Hospital of North Norway in Tromsø in the neighbor county to the north and St. Olavs Hospital in Trondheim to the south. The records were examined and the diagnoses were confirmed by a neurologist. Patients were included if they satisfied the criteria for clinically definite MS, laboratory supported definite MS, clinically probably MS or laboratory supported MS after the criteria described by Poser in 1983 [21] or MS after the criteria of McDonald from 2001 [22]. Cases that were misclassified or did not fulfill these criteria were excluded. Some patients had been treated at more than one hospital. By combining the files from different hospitals, additional information was obtained, and the diagnoses could be reconfirmed. We classified the cases as primary progressive MS (PPMS) or relapsing remitting MS (RRMS) course at onset. The stipulation of the year of the first symptom was based partly on older records of findings and symptoms in the files, and partly on the written information of the patients’ later recollection of former symptoms.

### Statistics

We have used the time of diagnosis as starting point for the epidemiological calculations. The point prevalence rate was defined as the proportion of the population in Nordland County with definitive or probable MS according to Poser or MS according to McDonalds at a specified time point. We calculated the point prevalence per 100 000 inhabitants for January 1, in 1980, 1990, 2000 and in 2010. The incidence rate was defined as the proportion of the population in Nordland County who got definitive or probable MS according to Poser or MS according to McDonalds during a specified time period. The average annual incidence was calculated for 5-year periods. Both prevalence and incidence were calculated for females and males separately, and we calculated the female to male (f/m) sex ratio.

We calculated the mean and median time delay from first symptom to diagnosis for each 5-year period. Data of population by age and gender were obtained from Statistics Norway [23]. Data considering the Sami population was obtained from the website of Sametinget, the Sami Parliament [24]. A Poisson distribution of the disease was assumed, and the 95% confidence interval (CI) for both the prevalence and the incidence was calculated. The age adjusted prevalence and incidence were calculated by the use of data from a standard European population [25]. A chi squared test was used to give a statistical description of the change of the proportion of PPMS in the prevalence rate over time. Statistical analyses were performed by the use of StatXact version 10 for Windows (Cytel Software, Cambridge, MA) and Microsoft Office Exel for Windows 7.

### Ethical approval

The study was approved by the Regional Committee for Medical and Health Research Ethics (REK Nord).

## Results

**Figure 2:**
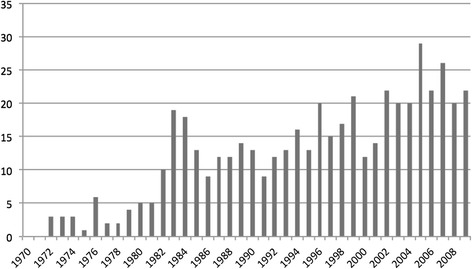
**Number of new cases diagnosed per year 1970 – 2009.**

We identified a total of 533 persons with definitive or probable MS. Ten persons had got the diagnoses before 1970, while 26 had received the diagnosis elsewhere and moved to the county. Twenty-two subjects diagnosed with MS had left the county, five had moved both to and from the county. Eighty patients had died during the period. The number of new cases diagnosed in the county is shown in Figure 2. In 7 cases the time of the first symptom could not be determined, 4 of these patients had got the diagnosis before 1970, 1 had got the diagnosis in the period 1975–79, 1 in the period 1985–89 and 1 in the period 90–94.

The number of persons with MS in the county in the present study differs slightly from the data previous published for the period 1970–2000 (20). Three cases were reclassified not to be MS. Seven persons received the diagnosis per letter after they had performed MRI at other hospitals, and were not registered in our files until they were treated in our department years later. Nineteen patients had been treated at the hospital in Mosjøen, a hospital that had not been included in the previous study. In the present study a total of 36 patients were identified exclusively outside our hospital.

On January 1, 2000 there were 158 persons diagnosed with MS that had not been examined with MRI. In all of the patients diagnosed during the period 2000–2009 an MRI had been performed.

### Prevalence

At January 1, 2010, 431 persons had definitive or probable MS. The crude prevalence was 182.4 per 100 000. The age adjusted prevalence was slightly lower, 174.4 per 100 000. The crude prevalence among women was 249.7 per 100 000 and among men 115.6 per 100 000. Table 1 shows the crude prevalence and prevalence by gender at the beginning of the decades. The prevalence increased significantly from 1970 to 2010, p < 0.001.

**Table 1:** **Prevalence of MS in Nordland County at the beginning of 4 decades**

		**Total**			
**Year**	**Population at risk**	**Cases**	**Prevalence (95% CI)**	**Age adjusted prevalence**	**Mean age (SD)**
1980	243 808	34	13.9 (9.7-19.5)	16.9	45.3 (10.7)
1990	239 532	152	63.5 (53.8-74.4)	70.5	45.4 (12.0)
2000	239 109	275	115.0 (101.8-129.4)	117.9	48.1 (11.6)
2010	236 271	431	182.4 (165.6-200.5)	174.4	51.2 (12.2)

**Figure 3:**
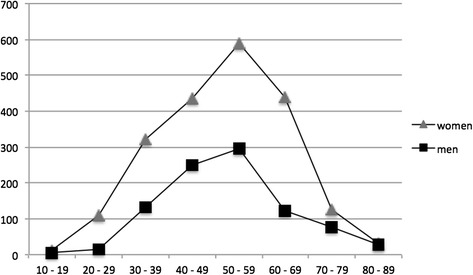
**Age-specific prevalence rates by gender on January 1, 2010.**

Figure 3 shows the age specific prevalence rates according to gender. The mean age of the MS patients increased continuously, and was 51.2 years in 2010. The female to male (f/m) prevalence ratio was 3.3 in 1980, but was then stable at 2.2 from 1990 to 2010 (Table 2).

**Table 2:** **Gender specific prevalences**

	**Women**		**Men**		**F/M sex ratio**
**Year**	**Cases**	**Prevalence**	**Cases**	**Prevalence**	
1980	26	21.6	8	6.5	3.3
1990	104	87.3	48	39.9	2.2
2000	189	158.1	86	71.9	2.2
2010	294	249.7	137	115.6	2.2

The proportion of PPMS in the prevalence numbers was 38.2% in 1980, and decreases continuously, to 18.6% in 2010 (Table 3). This is a statistical significant decline, p = 0.002.

**Table 3:** **Distribution of the prevalence of PPMS and RRMS course at onset**

**Year**	**PPMS, % (n)**	**RRMS, % (n)**	**Unknown, % (n)**
1980	38.2 (13)	52.9 (18)	8.8 (3)
1990	27.0 (41)	65.1 (99)	7.9 (12)
2000	21.5 (59)	76.0 (209)	3.3 (9)
2010	18.6 (80)	80.0 (345)	1.4 (6)

### Incidence

In the period 1970–2009 there were 497 persons who received the diagnosis while living in the county. The incidence was continuously increasing in the whole period, and the yearly average incidence was 10.1 per 100 000 in 2005–2009. This was a significant increase from the period 2000–2004, p = 0.031. The increase was also significant from 1985–1989 to 1995–1999, p = 0.031. The average annual incidences calculated in 5-year periods are shown in Table 4.

**Table 4:** **Yearly average incidence of MS in 5-year periods**

		**Total**		
**Years**	**Average population**	**Cases**	**Incidence (95% CI)**	**Age-adjusted incidence**
1970-1974	241867	9	0.7 (0.34-1.41)	0.9
1975-1979	242886	15	1.2 (0.69-2.04)	1.5
1980-1984	244614	57	4.7 (3.53-6.04)	5.3
1985-1989	241266	60	5.0 (3.80-6.40)	5.4
1990-1994	239953	63	5.3 (4.04-6.72)	5.4
1995-1999	240131	86	7.2 (5.73-8.85)	7.3
2000-2004	237783	88	7.4 (5.94-9.12)	7.6
2005-2009	235779	119	10.1 (8.36-12.98)	10.7

The mean age at the time of the diagnosis was stable during the whole period, and the time from first symptom to diagnosis was stable from 1975 to 2010 both in terms of mean and median (Table 5).

**Table 5:** **Age at diagnosis and time from first symptom to diagnosis**

	**Age at onset, year**		**Time from first symptom to diagnosis, years**			
**Years**	**Mean (SD)**	**Range**	**Mean (SD)**	**Median**	**Interquartil range**	**Range**
1970-1974	42.2 (8.7)	30-52	7.7 (5.9)	7	11	1-16
1975-1979	38.6 (10.5)	22-61	4.3 (5.3)	2,5	4.75	0-19
1980-1984	38.9 (11.8)	21-70	6.0 (7.9)	3	6	0-43
1985-1989	38.3 (11.1)	14-64	4.2 (5.9)	2	4.5	0-20
1990-1994	36.9 (10.4)	18-66	5.2 (6.5)	2	7	0-25
1995-1999	39.3 (10.0)	18-69	4.9 (5.6)	2,5	6	0-28
2000-2004	40.4 (11.4)	12-75	5.0 (6.1)	2	6	0-29
2005-2009	40.4 (11.0)	14-71	5.0 (6.1)	3	5.5	0-32

The f/m ratio was 2.1 in the period 1975–1979 and 2.2 in the period 2005–2009 (Table 6).

**Table 6:** **Gender specific incidence**

	**Women**		**Men**		**F/M sex ratio**
**Years**	**Cases**	**Incidence**	**Cases**	**Incidence**	
1970-1974	8	1.4	1	0.2	7.0
1975-1979	10	1.7	5	0.8	2.1
1980-1984	34	5.6	23	3.7	1.5
1985-1989	41	6.8	19	3.1	2.2
1990-1994	38	6.4	25	4.2	1.5
1995-1999	59	9.8	27	4.5	2.2
2000-2004	57	9.6	31	5.2	1.8
2005-2009	82	13.9	37	6.2	2.2

In the incidence numbers the proportion of PPMS decreased from 33.3% in 1970–1974 to 20.6% in 1990–1994. The proportion of PPMS was 21.8% of the new cases in the period 2005–2009, and had been quite stable from 1990 (Table 7).

**Table 7:** **Distribution of the incidence of PPMS and RRMS course at onset**

**Years**	**PPMS, % (n)**	**RRMS, % (n)**	**Unknown, % (n)**
1970-1974	33.3 (3)	55.6 (5)	11.1 (1)
1975-1979	33.3 (5)	66.7 (10)	0 (0)
1980-1984	28.8 (13)	70.2 (40)	7.0 (4)
1985-1989	26.7 (16)	66.7 (40)	6.7 (4)
1990-1994	20.6 (13)	77.8 (49)	1.6 (1)
1995-1999	17.4 (15)	82.6 (71)	0 (0)
2000-2004	19.3 (17)	79.5 (70)	1.1 (1)
2005-2009	21.8 (26)	78.2 (93)	0 (0)

## Discussion

The prevalence of MS has increased steadily in Nordland County during the last four decades. One contribution to this increase is the accumulation of cases. A total of 497 new patients were diagnosed, while only 80 died. The accumulation is reflected in the mean age, which increased from 45 to 51 years from 1980 to 2010. However, the main reason for the increase of prevalence is an increase of the incidence. The incidence was 0.7 per 100 000 in 1970–1974, and remained less than 5 per 100 000 until 1985. It reached 7 per 100 000 during 1995–1999, and was over 10 per 100 000 in the last period 2005–2010 (Table 4).

The Poser criteria were introduced in 1983, and we find a marked increase in new cases in 1983 and 1984 compared with previous years. In 2001 the diagnostic criteria of McDonald were established, again followed by an increase of diagnosed cases in our county. The new criteria probably made it easier to conclude in the diagnostic work-up. Also, following the introduction of new criteria more focus could have been put on the disease, and thereby increasing the diagnostic sensitivity. Because of the long time span of our study, we have included cases fulfilling the criteria either according to Poser or according to McDonald, or both. Other Norwegian studies were only Poser criteria are applied report increasing incidence [11,13]. Studies that compare the figures when Poser and McDonalds criteria are applied on the same population have found only small differences in the prevalence [26,27].

Magnetic resonance imaging (MRI) has become an important tool in the diagnostic work up of MS. The role of MRI in the study of MS-epidemiology and changes over time is an interesting but difficult issue. The first MRI scanner in Nordland was set up in autumn 2000. Prior to this, MRI was available in neighbouring regions, from 1986 in Trondheim and from 1991 in Tromsø. At prevalence day January 1, 2010 there were 5 MRI scanners in the county. Monosymptomatic cases and clinical isolated syndromes will not necessarily fulfill the criteria after Poser. If dissemination in time and space is demonstrated with MRI changes the diagnosis can be given with the McDonalds criteria. On January 1, 2000 there were 158 persons diagnosed with MS that had not performed MRI. All of the 207 patients diagnosed in the period 2000–2009 had performed a MRI scan. Of the 163 with RRMS, 38 were monosymptomatic at the time of diagnosis. The diagnosis was based on dissemination in time demonstrated on MRI. However, 21 of these 38 had a new clinical attack before 2010, thereby fulfilling the criteria for clinically definite MS after Poser as well. If those 17 not fulfilling the criteria for clinical MS after Poser were excluded, the prevalence rate at January 1, 2010 would drop from 182.4 to 175.2. This is a reduction of 3.9%. Although MRI findings are not included in the Poser criteria, the use of MRI will most likely increase the diagnostic sensitivity. Our experience is that if there are pathological findings on an MRI scan, more emphasis is put in the patient interview to reveal MS manifestations in the past. A patient with light symptoms or mainly sensory symptoms could be more easily diagnosed with this technique. On the other hand, MRI scans will rule out cases where other diagnoses are more likely.

Another potential contributor to increased diagnostics is the immunomodulatory treatment for MS that was available in clinical practice from the second half of the 90’s. It may have urged the necessity of diagnosing the condition, and increased the diagnostic sensitivity.

In the present study we report a steady age at time of diagnosis (Table 5). The mean age at onset, that is the time of diagnosis, was about 40 years during the whole period. We also report a steady time delay from the first symptom to the diagnosis. The mean time delay was 5.0 years in 2005–2009. This may intuitively seem a bit high, but is in accordance with other Norwegian studies. The results from a study in Oslo [11], also applying “time of diagnosis” as onset, are almost identical to ours. In 1972–1985 a mean age at onset of 38.7 year, and a mean time from the first symptom to the diagnosis of 5.9 year were found. This changed only slightly until 1985–1999, where the mean age at onset was 38.1, and the mean time from the first symptom to diagnosis was 5.2 year [11]. Other studies, which use first symptom as time of inclusion, report a marked decrease in time delay [12,13]. If we had used the “time of the first symptom” as starting point for the calculation, the mean time delay would have been 1.1 year and the median time delay 1 year in the period 2005–09. This is however an artificial time decline. When the “time of first symptom” is used, everyone included from the latest year would, by definition, have a time delay less than one year. We think that if increased incidence was only due to improved and faster diagnostic work up, the time delay from the first symptom to the diagnosis would be expected to decline.

Our findings show that the female to male (f/m) sex ratio has been stable over time. Based on the prevalence numbers it was 2.2 in 2010 which is unchanged from 1990 (Table 2). The f/m sex ratio based on incidence numbers was 2.1 in 1975–1979 and 2.2 in 2005–2009 (Table 6). Data from other high prevalence areas have also shown sex ratio stability over time [6,28], but across the world there has been an increase in the ratio [29]. For instance, a Canadian study of 27074 patients showed that the f/m ratio by year of birth increased over a period of at least 50 years [30]. In a Danish prevalence study including 9377 patients the f/m ratio increased from 1.31 in 1950 to 2.02 in 2005 [31]. A Swedish study including 8834 patients from the national MS patient registry did not find evidence for an increased f/m ratio by year of birth among MS patients born between 1931 and 1985 [32]. However, when the study was expanded to 19510 patients by including information from additional registers an increased sex ratio was identified. The f/m ratio was 1.70 among patients born 1931–1935, and increased to 2.67 among those borne 1981–1985 [33]. More than twice as many women than men have MS in Nordland, but as said this has been unchanged over the last decades. The data from our region is quite complete when it comes to registered patients in the given period, but we cannot exclude that the material is too small to demonstrate a trend.

We calculated the distribution of RRMS and PPMS both in the prevalence and the incidence numbers (Tables 3 and 7). We find a PPMS course of disease at onset in 18.6% of the prevalence at January 1, 2010. This is higher than other Norwegian studies performed after 2000, which find the proportion of PPMS ranging from 9.3 to 16.8% [12,13]. In a study from Finland, the proportion of PPMS was 22% in the period 1979–1993 [34]. We find that the proportion of PPMS has steadily decreased in the prevalence numbers from 1980 to 2010. However, the proportion of PPMS in the incidence numbers have been quite stable at approximately 20% from 1990–1994.

Findings from previous studies indicate a relatively low occurrence of MS in Troms and Finnmark, the two most northern counties of Norway, with a prevalence of 73.0 per 100 000 in 1993 [10]. The Sami is considered the traditionally indigenous inhabitants in northern Norway, Sweden, Finland and northwest Russia. It is a low prevalence of MS in the Sami population [19]. The majority lives in Finnmark, but there is no exact statistical data on the Norwegian Sami population. It is estimated that about 40 000 Sami are living in the country, of those are about 30 000 living north of the Arctic Circle. A clue about the distribution of the Sami population is the figures of the members in the Sami electoral register, in which membership is voluntary. At June 30, 2009 there were 13 890 persons registered in the Sami electoral register. In Nordland 1190 persons were registered. In Troms, with 156 494 inhabitants at 01.01.2010, 2807 persons were registered, while in Finnmark, with 72 856 inhabitants, as many as 7432 persons were registered in the electoral register. The high proportion of Sami could thus, at least to some extent, count for the low prevalence of MS in Troms and Finnmark compared to Nordland.

When prevalence and incidence rates are compared from different regions, it must be noticed that the surveys are from different times. The incidence and prevalence are in general increasing, and older studies will show lover occurrence than newer. The prevalence data from Troms and Finnmark are from 1993. The prevalence reported is lower than the prevalence in Nordland in 2000, but higher than the prevalence in Nordland in 1990. Nevertheless, from the same period (1995) the prevalence is much higher in Oslo to the south in the country. The present study is one of the two newest epidemiological studies in Norway, the other is from Vest-Agder to the very south. The prevalence in Nordland in 2010 is quite equal to that in Vest-Agder in 2007. It therefore seems to be a tendency towards a more homogeneous distribution of the disease in Norway. There are indications of dispersion of MS in Sweden. This change of distribution over time indicates that genetic background is not the only explanation of the origin of the disease [35].

There are certain strengths in our study that need to be pointed out. Our study has a long time span in a region with a relatively homogenous and stable population. Norway has a well-developed public healthcare, and it is likely that a person with symptoms of MS nowadays will have a prompt medical examination. In Nordland County, all patients with MS are treated by a neurologist employed by a public hospital. This suggests that the number of cases of MS from a hospital based survey, is very close to the real number in the population. In the present study, all medical files were re-evaluated by a neurologist to confirm the diagnosis. There are, however, some limitations. In the days when the diagnosis was made without the help of MRI, the diagnosis was more uncertain. Patients with mimicking symptoms could wrongly be diagnosed with MS, giving higher estimate of the occurrence. On the other hand, it is likely that the threshold to seek medical help has decreased in parallel to an increasing supply of health service, implying that benign MS and other light symptoms earlier could have been easily ignored both by the patient and the doctor giving a lower estimate of the prevalence.

## Conclusion

The occurrence of MS in Nordland County has been continuously increasing both in terms of prevalence and incidence over a period of 40 years. On January 1, 2010 the prevalence was 182.4 per 100 000. In the period 2005–2009 the average yearly incidence was 10.1 per 100 000. Nordland County is a high risk area for MS. Previous findings indicating a paradoxically low prevalence in the north of Norway could not be confirmed. Although the prevalence and incidence are higher among women then among men, we did not find an increased female to male ratio over time.
